# Aberrantly methylated genes in human papillary thyroid cancer and their association with *BRAF*/*RAS* mutation

**DOI:** 10.3389/fgene.2013.00271

**Published:** 2013-12-05

**Authors:** Yasuko Kikuchi, Eiichi Tsuji, Koichi Yagi, Keisuke Matsusaka, Shingo Tsuji, Junichi Kurebayashi, Toshihisa Ogawa, Hiroyuki Aburatani, Atsushi Kaneda

**Affiliations:** ^1^Genome Science Division, Research Center for Advanced Science and Technology, The University of TokyoTokyo, Japan; ^2^Department of Metabolic Care and Endocrine Surgery, Graduate School of Medicine, The University of TokyoTokyo, Japan; ^3^Department of Pathology, Graduate School of Medicine, The University of TokyoTokyo, Japan; ^4^Department of Breast and Endocrine Surgery, Kawasaki Medical UniversityOkayama, Japan; ^5^Department of Molecular Oncology, Graduate School of Medicine, Chiba UniversityChiba, Japan; ^6^CREST, Japan Science and Technology AgencySaitama, Japan

**Keywords:** DNA methylation, thyroid cancer, CIMP (CpG island methylator phenotype), BRAF, RAS, oncogene mutation

## Abstract

Cancer arises through accumulation of epigenetic and genetic alteration. Aberrant promoter methylation is a common epigenetic mechanism of gene silencing in cancer cells. We here performed genome-wide analysis of DNA methylation of promoter regions by Infinium HumanMethylation27 BeadChip, using 14 clinical papillary thyroid cancer samples and 10 normal thyroid samples. Among the 14 papillary cancer cases, 11 showed frequent aberrant methylation, but the other three cases showed no aberrant methylation at all. Distribution of the hypermethylation among cancer samples was non-random, which implied existence of a subset of preferentially methylated papillary thyroid cancer. Among 25 frequently methylated genes, methylation status of six genes (*HIST1H3J, POU4F2, SHOX2, PHKG2, TLX3, HOXA7*) was validated quantitatively by pyrosequencing. Epigenetic silencing of these genes in methylated papillary thyroid cancer cell lines was confirmed by gene re-expression following treatment with 5-aza-2′-deoxycytidine and trichostatin A, and detected by real-time RT-PCR. Methylation of these six genes was validated by analysis of additional 20 papillary thyroid cancer and 10 normal samples. Among the 34 cancer samples in total, 26 cancer samples with preferential methylation were significantly associated with mutation of *BRAF*/*RAS* oncogene (*P* = 0.04, Fisher's exact test). Thus, we identified new genes with frequent epigenetic hypermethylation in papillary thyroid cancer, two subsets of either preferentially methylated or hardly methylated papillary thyroid cancer, with a concomitant occurrence of oncogene mutation and gene methylation. These hypermethylated genes may constitute potential biomarkers for papillary thyroid cancer.

## Introduction

Papillary thyroid cancer is the most common cancer derived from follicular cells. It is estimated that approximately 23,500 cases of differentiated thyroid cancer occur per year in the United States (Jemal et al., 2005), and 19,000 papillary thyroid cancer cases per year in the European Union (http://globocan.iarc.fr/). In Japan, about 8000 patients suffer from thyroid cancer every year, 80% of which are papillary cancer. While prognosis for papillary thyroid cancer is generally good, with a 10-year survival rate above 90%, some patients die of distant metastases and/or repeated recurrence (Ezaki et al., 1992; Yamashita et al., 1998).

RET/PTC (Rearranged in Transformation/Papillary Thyroid Carcinoma) re-arrangement, *BRAF* (V-Raf murine sarcoma viral oncogene homolog B) and *RAS* (Rat Sarcoma viral oncogene homolog) point mutations are frequently observed in papillary thyroid cancer (Mitsutake et al., 2006; Knauf and Fagin, 2009). Mutation of T to A at 1799 in the exon 15 of the *BRAF* gene has been reported in 28–69% of papillary thyroid cancer cases, while point mutations of *RAS* genes are detected in 5–20% (Cohen et al., 2003; Kimura et al., 2003; Namba et al., 2003; Kondo et al., 2007). Papillary thyroid cancer with poor prognosis is associated with *BRAF* mutation (Xing et al., 2005; Lee et al., 2012), whereas concomitantly, lengthy disease-free interval is not (Ulisse et al., 2012).

Patients with papillary thyroid cancer are generally treated by surgery. But it is difficult to decide whether total thyroidectomy, hemithyroidectomy, prophylactic central neck dissection or no dissection, should be performed in patients without preoperative or intraoperative evidence of metastatic lymph nodes (Xing et al., 2013). Association of *BRAF* mutation with occult central neck lymph node metastases (Joo et al., 2012) might support use of *BRAF* mutation as an indication for prophylactic central neck dissection for patients with conventionally low- to intermediate-risk papillary thyroid cancer. But precise diagnosis to define prognosis and suitable therapy is currently impossible. Molecular biomarkers would therefore simplify disease management (McLeod et al., 2013).

Along with genetic alterations, accumulation of epigenetic alterations is known to affect cancer development (Baylin and Ohm, 2006; Feinberg et al., 2006; Esteller, 2007). Aberrant DNA methylation at promoter regions is a major epigenetic alteration to silence tumor suppressor genes in many cancer types. *RASSF1A* (Ras association (RalGDS/AF-6) domain family member 1) is methylated in 20% of papillary thyroid cancer, leading to activation of the RAS-MAPK (Mitogen-Activated Protein Kinase) signal (Xing et al., 2004). Papillary thyroid cancer is also reported to involve methylation of other genes, including *RARB* (Retinoic Acid Receptor, Beta), *p16*^INK4A^ (*CDKN2A*, Cyclin-Dependent Kinase Inhibitor 2A), *TSHR* (Thyroid Stimulating Hormone Receptor), *CDH1* (Cadherin 1, type 1, E-cadherin), *DAPK* (Death-Associated Protein Kinase 1), and *MLH1* (mutL Homolog 1) (Hoque et al., 2005; Guan et al., 2008; Mohammadi-asl et al., 2011). While a few genes known to be aberrantly methylated in other cancers were analyzed in these studies, methylation frequencies ranged from 15 to 33%. Involvement of genes in aberrant DNA methylation, however, has not been well-clarified in papillary thyroid cancer. Whether any subset of papillary thyroid cancer shows preferential aberrant methylation, and whether such methylation and other clinicopathological factors are associated are also unclear.

We here analyzed DNA methylation status of promoter regions on a genome-wide scale, using the Illumina Infinium HumanMethylation27 BeadChip technique on 14 clinical papillary thyroid cancer samples and 10 normal thyroid samples. For genes frequently hypermethylated in cancer, methylation status was validated quantitatively by pyrosequencing, using 20 additional clinical cancer samples and 10 additional normal samples. Methylation-associated gene silencing was confirmed by gene re-expression following 5-aza-2′-deoxycytidine and trichostatin A treatment, and by quantitative reverse transcription-polymerase chain reaction (RT-PCR) on thyroid cancer cell lines. We found a number of genes with frequent aberrant methylation and silencing in papillary thyroid cancer, and a subset of cancer with preferential aberrant methylation.

## Materials and methods

### Clinical samples and cell lines

We obtained 79 primary papillary thyroid cancer samples from patients who underwent thyroidectomy at The University of Tokyo, with written informed consent. These samples were immediately frozen with liquid nitrogen and stored at −80°C. The frozen materials were microscopically examined for cancer cell contents by pathologists and were dissected to enrich cancer cells when necessary. Thirty-four cancer samples containing more than 40% of cancer cells were selected and used for further analysis. DNA was extracted by QIAamp DNA Micro Kit (QIAGEN, Valencia, CA). Thyroid cancer cell line BHT-101 was obtained from DSMZ (Leibniz Institute DSMZ-German Collection of Microorganisms and Cell Cultures), TPC-1 was provided from Dr. Mitsutake, University of Nagasaki (Ishizaka et al., 1989) and KTC-1 and KTC-3 cell lines were provided from Dr. Kurebayashi, Kawasaki Medical University (Kurebayashi et al., 2000, 2006). These cell lines were maintained in RPMI 1640 (Gibco, Grand Island, NY) supplemented with 10% fetal bovine serum, streptomycin sulfate (100 mg/L), and penicillin G sodium (100 mg/L). Peripheral blood cell samples were purchased from The Coriell Cell Repositories. This research was certified by the Ethics Committee of The University of Tokyo and Chiba University.

### Infinium assays

High-resolution methylation analysis was conducted on the Illumina Infinium HumanMethylation27 microarray platform. This BeadChip assay measures methylation, given as a β-value, at more than 27,000 CpG loci covering 15,000 genes. For each CpG site, the β-value is the ratio of the fluorescence signal from the methylated probe over the sum of methylated and unmethylated probe signals. The β-value, ranging from 0.00 to 1.00, reflects the methylation level of the individual CpG site. Bisulfite conversion, whole-genome amplification, labeling, hybridization, and scanning were carried out according to the manufacturer's protocols. According to the previously proposed classification (Weber et al., 2007), Infinium probes were classified into three categories: high-CpG, intermediate-CpG, and low-CpG probes, on the basis of CpG ratio (the ratio of observed CpG count over expected CpG count) and GC contents within 500 bp region around the probe site (Matsusaka et al., 2011). Genes in X and Y chromosomes were excluded to avoid gender differences. Infinium enables us to analyze DNA methylation levels systematically for more than 14,000 genes, but methylation level of a single CpG site detected by Infinium may not precisely represent methylation status of promoter CpG island. Some Infinium probes might be less quantitative; in analysis of control samples with known levels of methylation (0, 25, 50, 75, 100%), the observed β-values generally correlated with the expected β-values, while some probes showed lower β-values (0.0–0.3) for 75% control or higher β-values (0.7–1.0) for 25% control (Nagae et al., 2011).

### Pyrosequencing analysis

Quantitative validation for methylation status was carried out by pyrosequencing as previously reported (Matsusaka et al., 2011). Primers were designed to include no or only one CpG site in a primer sequence using Pyro Q-CpG Software (QIAGEN), to amplify bisulfite-treated DNA regions containing several CpG sites. For C of CpG site within a primer sequence, a nucleotide which does not anneal to C or U was chosen, e.g., adenosine (A). Briefly, the biotinylated PCR product was bound to streptavidin sepharose beads HP (Amersham Biosciences, Sweden), washed, and denatured using a 0.2 mol/L NaOH solution. After addition of 0.3 μmol/L sequencing primer to the purified, single-stranded PCR product, pyrosequencing was carried out using PyroMark Q96 ID System (QIAGEN) according to the manufacturer's instructions. Primer sequences and conditions for *HIST1H3J* (Histone cluster 1, H3j), *POU4F2* (POU class 4 homeobox 2), *SHOX2* (Short stature homeobox 2), *PHKG2* (Phosphorylase Kinase, Gamma 2), *TLX3* (T-cell Leukemia homeobox 3), and *HOXA7* (Homeobox A7), are shown in Table 1. Control samples with known levels of methylation (0, 25, 50, 75, 100%) were prepared as previously described (Yagi et al., 2010). Pyrosequencing is not systematic, but highly quantitative (Matsusaka et al., 2011), and enables us to precisely validate the methylation level at one CpG site, as determined by the Infinium assay, as well as at surrounding CpG sites. Mean methylation levels of these CpG sites were calculated to represent methylation status of each gene promoter, and were displayed in figures by heatmap or dot chart.

**Table 1:** **Primer sequences for pyrosequencing of six potential methylation biomarkers**.

**Genes**	**Primer sequences for PCR (Forward/Reverse)**		**Sequencing primers**
*HIST1H3J*	F	GTTATAAATTTTGGTAGAAGTTATTGT	ATGGTTAGGAAGAAGTAGATAGT
	R^*^	ACCTTAATAACCAACTACTTCC	
*POU4F2*	F	GGGGAGAGGGGAGTATAA	ATTAGTTTAGATTGATAGTAGAGG
	R^*^	AAAAAAAACTATACCAAATTAAACTCACCC	
*SHOX2*	F^*^	TTGGGGGGGTTGGAGTAGTAAA	AACCCCCTAAATTCTTCCAT
	R	CTCCTTCTTCTCCTTCACTTTCTAATC	
*PHKG2*	F	GTTTGTAATTTTAGTATTTTGGGAGGTTGA	AAGTTTAAGGTTGTAGTGA
	R^*^	TCCCTAACTAAATTCAACATTTTCTCTT	
*TLX3*	F	TGGTTGAGGTAGGAGAGGAATTAGTA	GGTTTAAGAAAGATGATATAGAG
	R^*^	CACTAAAACTTTACCAAAAATAC	
*HOXA7*	F^*^	GGGAGTAAAGGAGTAAGAAGT	CAACAAATCACAAATCAAAATTA
	R	ACCCAACAACAAATCACAAATCAAAATT	

* Primers with 5 ′-biotin tag.

#### Mutation analysis

Mutations at *BRAF* 1799, *KRAS* (Kirsten Rat Sarcoma viral oncogene homolog) 34, 35, 38, *NRAS* (Neuroblastoma RAS viral oncogene homolog) 181, 182, 183, *HRAS* (Harvey Rat Sarcoma viral oncogene homolog) 35, 37, 181, 182, 183, were analyzed by genotyping assays on MassARRAY platform, by detecting mass difference of the extended nucleotide using matrix assisted laser desorption ionization-time of flight-mass spectrometry (MALDI-TOF-MS) (Yagi et al., 2012). First, PCR amplification primers and a post-PCR extension primer were designed using MassARRAY Assay Design 3.0 software (Sequenom), and listed in Table 2. Those mutations were analyzed in a single reaction by multiplex PCR. PCR amplification was performed in 5-μL volume containing 0.5 unit of Taq polymerase, 5 ng of genomic DNA, 0.5 pmol of PCR primer and 2.5 nmol of dNTPs. PCR reactions were cycled at 94°C for 15 min, followed by 45 cycles of 94°C for 20 s, 56°C for 30 s and 72°C for 1 min. Shrimp alkaline phosphatase treatment was performed at 37°C for 20 min and 85°C for 5 min. Post-PCR primer extension was carried out using 5.6 pmol of extension primer. Extension reaction was cycled at 94°C for 30 s, followed by 40 cycles of 94°C for 5 s, 5 cycles of 52°C for 5 s and 80°C for 5 s, and 72°C for 3 min. Reaction products were transferred to a SpectroCHIP (Sequenom) and mass difference was analyzed using MALDI-TOF-MS. Irradiation of the matrix-oligonucleotide-cocrystal with a laser beam ultimately results in desorption and ionization of the oligonucleotides, which then can be accelerated in an electrical field into the TOF device. The TOF device separates the accelerated analyte ions of different mass-to-charge (m/z) ratios by providing a field-free drift tube of defined length. After passing through the tube, ions are detected; every signal is assigned to a specific molecular mass calculated from the TOF. The extended bases at possible mutation sites are determined from the difference of nucleotide molecular masses (Vogel et al., 2009).

**Table 2:** **Primer sequences used for mutation analysis (MALDI-TOF-MS assays)**.

**Mutation sites**	**Primer sequences (Forward/Reverse)**		**Extend primers**
*BRAF*_1799	F	ACGTTGGATGTTCAAACTGATGGGACCCAC	TGATTTTGGTCTAGCTACAG
	R	ACGTTGGATGTCTTCATGAAGACCTCACAG	
*HRAS*_34	F	ACGTTGGATGAATGGTTCTGGATCAGCTGG	ACTCTTGCCCACACCGC
	R	ACGTTGGATGGACGGAATATAAGCTGGTGG	
*HRAS*_35	F	ACGTTGGATGAATGGTTCTGGATCAGCTGG	AGCGGGTGGTGGTGGGCGCCG
	R	ACGTTGGATGGACGGAATATAAGCTGGTGG	
*HRAS*_37	F	ACGTTGGATGAATGGTTCTGGATCAGCTGG	TCATCGCACTCTTGCCCACAC
	R	ACGTTGGATGGACGGAATATAAGCTGGTGG	
*HRAS*_38	F	ACGTTGGATGAATGGTTCTGGATCAGCTGG	CAGCGCACTCTTGCCCACA
	R	ACGTTGGATGGACGGAATATAAGCTGGTGG	
*HRAS*_181	F	ACGTTGGATGTGGCAAACACACACAGGAAG	CATGGCGCTGTACTCCTCCT
	R	ACGTTGGATGTGTTGGACATCCTGGATACC	
*HRAS*_182	F	ACGTTGGATGTGGCAAACACACACAGGAAG	CATGGCGCTGTACTCCTCC
	R	ACGTTGGATGTGTTGGACATCCTGGATACC	
*HRAS*_183	F	ACGTTGGATGTGGCAAACACACACAGGAAG	CGCATGGCGCTGTACTCCTC
	R	ACGTTGGATGTGTTGGACATCCTGGATACC	
*KRAS*_34	F	ACGTTGGATGTAGCTGTATCGTCAAGGCAC	ACTCTTGCCTACGCCAC
	R	ACGTTGGATGAGGCCTGCTGAAAATGACTG	
*KRAS*_35	F	ACGTTGGATGTAGCTGTATCGTCAAGGCAC	CTGTGGTAGTTGGAGCTG
	R	ACGTTGGATGAGGCCTGCTGAAAATGACTG	
*KRAS*_37	F	ACGTTGGATGTAGCTGTATCGTCAAGGCAC	GAGGGGCACTCTTGCCTACGC
	R	ACGTTGGATGAGGCCTGCTGAAAATGACTG	
*KRAS*_38	F	ACGTTGGATGTAGCTGTATCGTCAAGGCAC	AGGCACTCTTGCCTACG
	R	ACGTTGGATGAGGCCTGCTGAAAATGACTG	
*NRAS*_181	F	ACGTTGGATGTCGCCTGTCCTCATGTATTG	ATACTGGATACAGCTGGA
	R	ACGTTGGATGCCTGTTTGTTGGACATACTG	
*NRAS*_182	F	ACGTTGGATGTCGCCTGTCCTCATGTATTG	ATGGCACTGTACTCTTCT
	R	ACGTTGGATGCCTGTTTGTTGGACATACTG	
*NRAS*_183	F	ACGTTGGATGTCGCCTGTCCTCATGTATTG	CTGGATACAGCTGGACA
	R	ACGTTGGATGCCTGTTTGTTGGACATACTG	

### 5-aza-2′-deoxycytidine and trichostatin a treatment

Thyroid cancer cell lines were cultured at a density of 3 × 10^5^ cells/10-cm dish on Day 0. Cells were exposed to 3 μM 5-aza-2′-deoxycytidine (Sigma-Aldrich, St. Louis, MO) on Days 1, 2, and 3, and to 300 nM trichostatin A (Sigma-Aldrich) on the Day 3. 5-Aza-2′-deoxycytidine induces hypomethylation of DNA by inhibiting DNA methyltransferase, and re-expression of silenced genes by 5-aza-2′-deoxycytidine treatment is synergistically enhanced by trichostatin A, a histone deacetylase inhibitor (Suzuki et al., 2002). 5-Aza-2′-deoxycytidine is unstable in aqueous solution, and thus a 20 mM solution in dimethyl sulfoxide (DMSO) was freshly prepared, and diluted in medium to 3 μM every day immediately before medium change. The medium was changed every 24 h, and the cells were harvested on Day 4.

### Quantitative PCR analysis

RT-PCR was performed using CFX96 Touch TM Real-Time PCR Detection System (BIORAD Laboratories). cDNA was synthesized from 1 μg of total RNA treated with DNase I with a Superscript III kit (Invitrogen, Life Technologies). The quantity of cDNA of each gene in a sample was measured by comparing it with standard samples that contained 10^1^ to 10^6^ copies of the genes, and normalized to that of *PPIA* (Peptidylprolyl Isomerase A). Primer sequences are shown in Table 3.

**Table 3:** **Primer sequences for real-time RT-PCR in gene re-expression analysis**.

**Gene**	**Primer sequences (Forward/Reverse)**		**Anneal (°C)**	**Product (bp)**
*HIST1H3J*	F	AAATCAAGCAGAGGCGAAGTCGGA	58	106
	R	GGATAGTGGGTCTCGTCAAAAAGC		
*POU4F2*	F	CACCAAGCCTGAACTCTTCAAT	58	101
	R	GCTGAATGGCAAAGTAGGCTTCG		
*SHOX2*	F	AAATCAAGCAGAGGCGAAGTCGGA	58	85
	R	GGATAGTGGGTCTCGTCAAAAAGC		
*PHKG2*	F	TGATCTTGTTCACACTCCTGGCT	58	145
	R	GAGATCAGGTCTTTGACAGTGCT		
*TLX3*	F	CTGTCTGCACAACTCGTCACTCTT	60	79
	R	GACAGCGGGAACCTTGGAACTATC		
*HOXA7*	F	AGTTCCACTTCAACCGCTACCTGAC	58	131
	R	GTCCTTATGCTCTTTCTTCCACTTC		

### Statistical analysis

P-values were calculated to compare methylation(+) cancer and methylation(−) cancer and to analyze the correlation of the methylation status to clinicopathological features. Fisher's exact test was used for analysis of binary features such as sex, distant metastasis, recurrence, and mutation of *BRAF*/*RAS* oncogenes (with simple choice between male and female, occurrence and no occurrence); *t*-test was used for more descriptive features that do not imply a choice, such as age, tumor size, number of lymph nodes with metastasis, and thyroglobulin. When *P* < 0.05, the correlation was considered statistically significant. *P*-values were also calculated by *t*-test to compare distribution of methylation ratios between cancer and normal samples. When *P* < 0.05, the difference of the methylation ratios between cancer and normal samples was considered statistically significant. The dot chart and heatmap were drawn using Excel software and Java TreeView software (http://jtreeview.sourceforge.net/).

## Results

### Oncogene mutation status in papillary thyroid cancer

**Figure 1:**
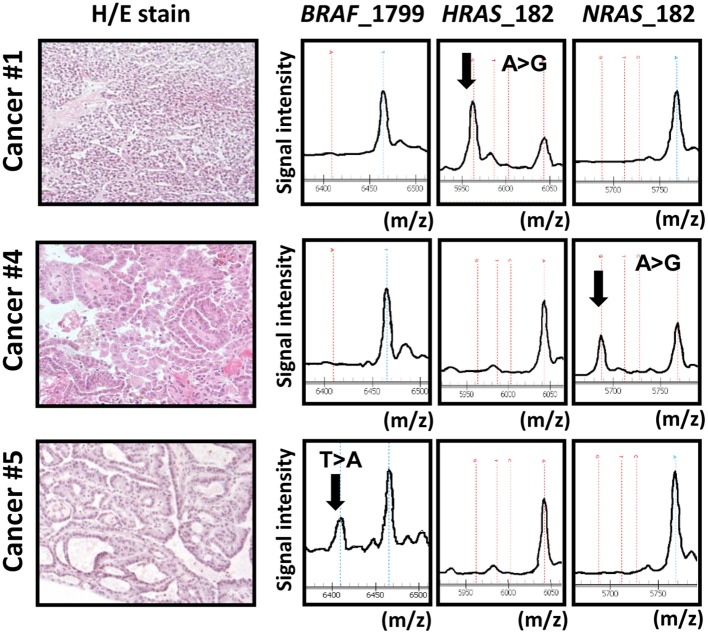
**Representative data obtained from MALDI-TOF-MS imaging for three cancer samples**. Cancer samples #1, #4, and #5 correspond to papillary cancer samples #1, #4, and #5 in Figure 3. First column: photomicrograph of a tumor section with hematoxylin/eosin (H/E) staining. Second, third, and fourth columns: MALDI-TOF-MS profile with detection of *BRAF*/*RAS* mutations. *X*-axis indicates mass-to-charge ratio (m/z) to distinguish wild-type and mutant allele, and Y-axis indicates signal intensity.

We analyzed mutation status of *BRAF* and *RAS* (*HRAS*, *NRAS*, and *KRAS*) oncogenes in 34 papillary thyroid cancer samples using MALDI-TOF-MS (Figure 1). *BRAF* mutation was detected in 67% (23/34) of the 34 samples, whereas *HRAS*, *NRAS*, and *KRAS* mutations were detected less frequently, in 3% (1/34), 3% (1/34), and 0% (0/34) sample, respectively. Each oncogene mutation was mutually exclusive; 25 among the 34 samples (75%) were oncogene-mutation(+) cancer.

### DNA methylation analysis using illumina infinium beadarray

**Figure 2:**
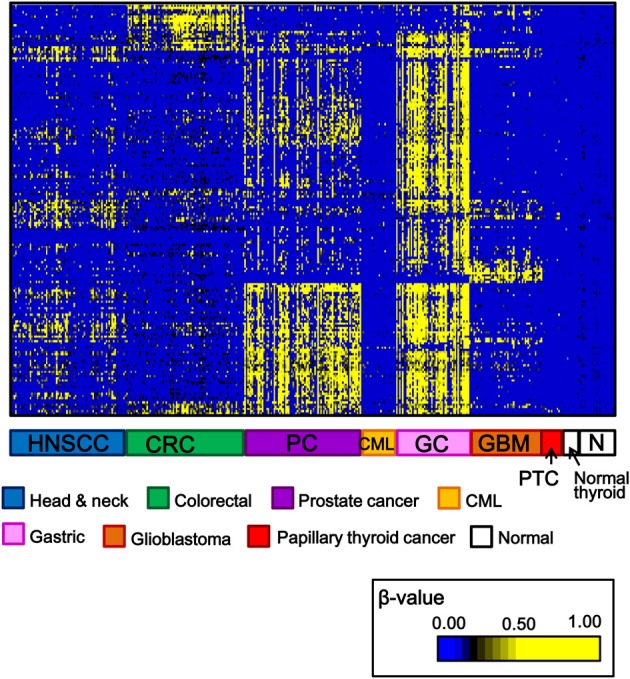
**Heatmap for methylation status of several cancer types**. Genes with β-value <0.17 in normal samples and with standard deviation of β-value in cancer samples >0.15, were selected and their β-values were shown by heatmap. Each cancer type showed different methylation profile, and papillary thyroid cancer displayed fewer aberrantly methylated genes than other cancer types. HNSCC, head and neck squamous cell carcinoma; CRC, colorectal cancer; PC, prostate cancer; CML, chronic myeloid leukemia; GC, gastric cancer; GBM, glioblastoma; PTC, papillary thyroid cancer; N, normal samples corresponding to these cancer types.

Among 34 papillary thyroid cancer and 24 normal thyroid samples, 14 and 10 samples, respectively, were analyzed using Infinium 27K BeadArray. Methylation data of other cancer types (80 head and neck squamous cell cancers, 50 gastric cancers, 80 colorectal cancers, 80 prostate cancers, 24 chronic myeloid leukemias, 50 glioblastomas), and normal samples of corresponding tissues were collected from National Center for Biotechnology Information, Gene Expression Omnibus (http://www.ncbi.nlm.nih.gov/gds: GSE25089 for head and neck squamous cell carcinoma, GSE31789 for gastric cancer, GSE27130 for colorectal cancer, GSE26126 for prostate cancer, GSE31600 for chronic myeloid leukemia, and GSE22867 for glioblastoma). To analyze aberrantly methylated genes in cancer samples, probes with β-value < 0.17 in all the normal samples and with standard deviation of β-values in cancer samples >0.15 were selected, and shown in Figure 2. Each cancer type including papillary thyroid cancer showed a unique pattern of aberrant promoter methylation.

### Aberrantly methylated genes in papillary thyroid cancer

**Figure 3:**
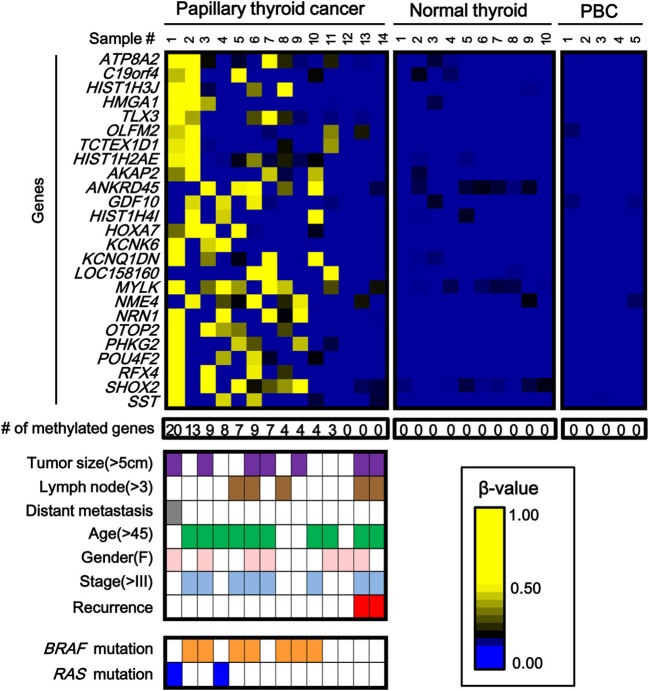
**Aberrantly methylated genes frequently observed in papillary thyroid cancer**. PBC, peripheral blood cells. We detected 25 genes that were hypermethylated (β > 0.25) in at least three of 14 papillary thyroid cancer samples, but not methylated (β < 0.2) in 10 normal samples. Among the 14 papillary cancer samples, 11 showed aberrant methylation in three or more genes, whereas the other three samples had no aberrant methylation. Purple, tumor size; brown, number of metastatic lymph nodes >3; gray, distant metastasis(+); green, age over 45 years old; pink, female; pale blue, stage III or IV; red, recurrence (+); orange, *BRAF* mutation(+); blue, *RAS* mutation (+). The two samples with recurrence (red) had no aberrant methylation (*P* = 0.03, Fisher's exact test). *BRAF*/*RAS* oncogene mutations were all observed in methylation(+) samples (*P* = 0.03, Fisher's exact test).

While the number of aberrantly methylated genes was relatively small in papillary thyroid cancer (Figure 2), 25 genes showed frequent hypermethylation (β > 0.25) in three or more samples among the 14 papillary thyroid cancer samples, and no methylation (β < 0.2) in all the 10 normal samples (Figure 3). To check that the hypermethylation status was not due to contaminated blood cells, the methylation status of these genes in peripheral blood cells was also analyzed to see that none of them were methylated in blood (Figure 3).

Among 14 papillary cancer samples, 11 samples showed aberrant methylation in three or more genes, whereas the other three samples showed no aberrant methylation at all (Figure 3). When methylation status was compared with clinicopathological factors, the two cancer cases with recurrence were both methylation-negative (*P* = 0.03, Fisher's exact test) (Figure 3). Nine of the 11 frequently methylated samples showed mutation of *BRAF*/*RAS* oncogenes, whereas none of the three methylation-negative samples showed oncogene mutation (*P* = 0.03, Fisher's exact test). Other clinicopathological factors, including tumor size, lymph node metastasis, distant metastasis, tumor stage, age, or sex, did not show significant difference.

**Figure 4:**
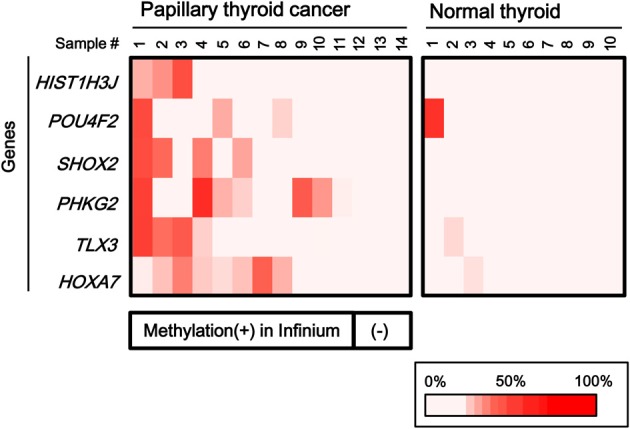
**Confirmation of aberrant methylation by pyrosequencing**. Six genes were randomly chosen among the 25 frequently methylated genes, and their methylation status was quantitatively validated by pyrosequencing, using the 14 papillary thyroid cancer samples and 10 normal thyroid samples that were analyzed by Infinium. Although one normal sample showed high methylation in *POU4F2*, aberrant methylation of the six genes in the papillary cancer samples was confirmed. **Bottom**, the corresponding data for aberrant methylation obtained by Infinium analysis (Figure 3).

To validate the methylation status of these genes, six out of the 25 genes, *HIST1H3J*, *POU4F2*, *SHOX2*, *PHKG2*, *TLX3*, and *HOXA7*, were randomly chosen and analyzed by pyrosequencing, a highly quantitative method (Figure 4). Although one normal sample showed high methylation in *POU4F2*, frequent hypermethylation of these genes in papillary cancer samples was confirmed, while normal thyroid samples were generally unmethylated.

### Evaluation of gene silencing

**Figure 5:**
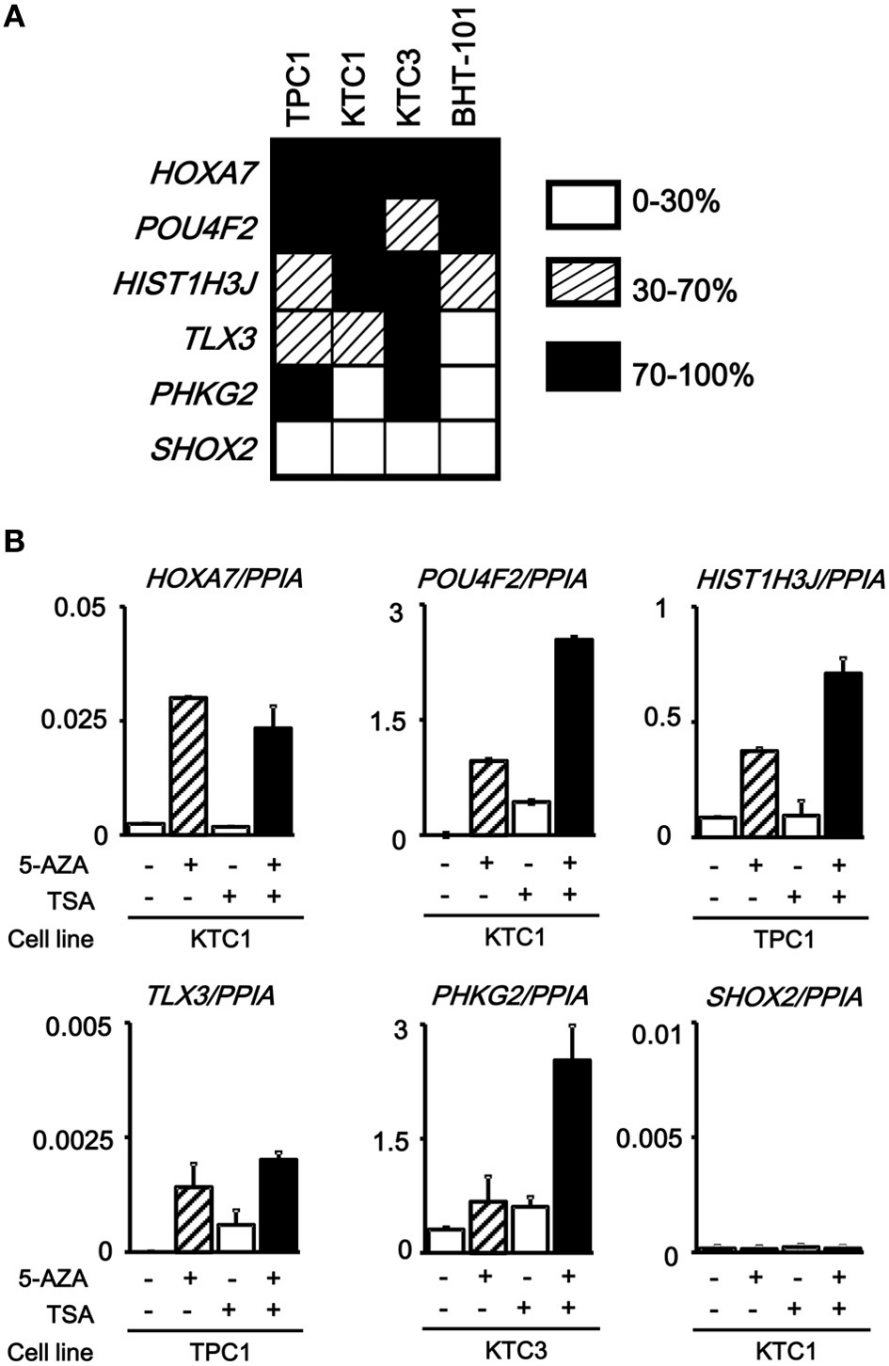
**Silencing of aberrantly methylated genes. (A)** Methylation status of the *HOXA7*, *POU4F2*, *HIST1H3J*, *TLX3*, *PHKG2*, *SHOX2* genes was analyzed in papillary thyroid cancer cell lines TPC1, KTC1, and KTC3 and anaplastic thyroid cancer cell line BHT-101 by pyrosequencing. *Open box*, 0–30% methylation, presumably no allele methylation. *Hatched box*, 30–70% methylation, presumably hemi-allelic or partial methylation. *Closed box*, 70–100% methylation, presumably bi-allelic and dense methylation. All the genes except *SHOX2* showed dense methylation in at least one papillary thyroid cancer cell line. **(B)** Real-time RT-PCR analysis of the methylated genes. Cells were treated with 5-aza-2′-deoxycytidine (5-AZA) and/or trichostatin A (TSA). Gene expression levels were normalized to that of *PPIA* (*Peptidylprolyl Isomerase A*, or *cyclophilin A*). All the genes except *SHOX2* showed no or very low expression in the analyzed cancer cell line, and showed re-expression in cells treated with 5-AZA/TSA.

The analyzed tissue samples include a part of non-tumor cells (see Materials and Methods). To evaluate whether these aberrantly methylated genes were silenced in cancer cells, we analyzed methylation status of these six genes in papillary thyroid cancer cell lines (TPC1, KTC1, and KTC3) and anaplastic thyroid cancer cell line BHT-101 (Figure 5A). All the genes except *SHOX2* showed dense methylation in at least one papillary thyroid cancer cell line, confirming that hypermethylation detected in cancer tissue samples is due to hypermethylation in cancer cells.

We next performed real-time RT-PCR for the six genes. All the genes except *SHOX2* showed no or very low expression in the analyzed, methylated cancer cell line, and showed re-expression in cells treated with 5-aza-2′-deoxycytidine and/or trichostatin A (Figure 5B). *SHOX2* was neither expressed, nor methylated in KTC1 (Figure 5A). Consequently, its expression was not reversed by the deoxycytidine/trichostatin treatment (Figure 5B). This is presumably because *SHOX2* was silenced in KTC1 by mechanisms other than promoter methylation, e.g., by depletion of appropriate transcription factors.

### Methylation analysis of the six genes in additional samples

**Figure 6:**
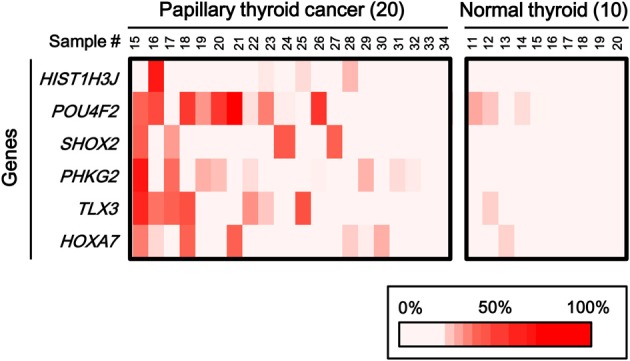
**Aberrant methylation of *HOXA7*, *POU4F2*, *HIST1H3J*, *TLX3*, *PHKG2*, *SHOX2* genes in additional samples: 20 cancer samples and 10 normal ones**. Methylation status of the six genes was confirmed by pyrosequencing. Bottom, color scale with known methylation degrees (0–100%).

To statistically extend the validation of aberrant methylation of the six genes, we analyzed the methylation status in 20 additional papillary thyroid cancer samples and 10 additional normal thyroid samples by pyrosequencing (Figure 6). A similar fraction of cancer samples showed high methylation in each gene (2/20 for *HIST1H3J*, 8/20 for *POU4F2*, 4/20 for *SHOX2*, 5/20 for *PHKG2*, 6/20 for *TLX3*, and 4/20 for *HOXA7*).

**Figure 7:**
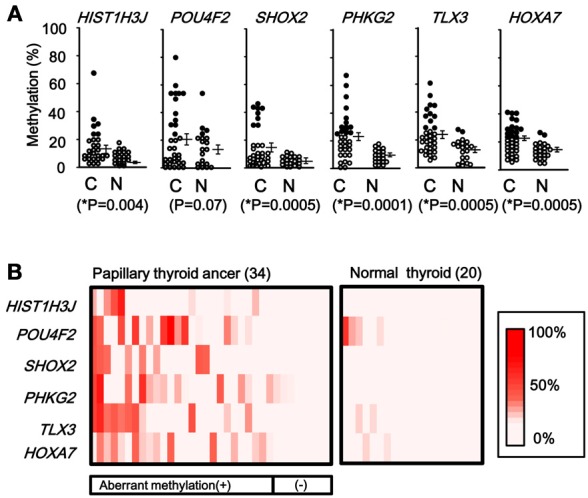
**Methylation status of the six genes in 54 samples analyzed by pyrosequencing. (A)** Each gene was methylated to a different extent in each sample. *C*, 34 cancer samples. *N*, 20 normal samples. *Circle*, methylation ratio of each sample. Filled circles indicate that the gene is methylated with the methylation ratio >25%. *Bars*, the mean and standard error of methylation ratios. *P*-values were calculated by *t*-test to compare distribution of methylation ratios between cancer (*C*) and normal (*N*) samples. Five genes (*HIST1H3J*, *SHOX2*, *PHKG2*, *TLX3*, and *HOXA7*) showed significantly higher methylation in cancer (*P* < 0.05, *t*-test). *POU4F2* tended to show higher methylation in cancer (*P* = 0.07, *t*-test). **(B)** Heatmap for methylation status of the 54 analyzed samples. Among the 34 papillary thyroid cancer samples, 26 were aberrant methylation(+) in at least one of six genes, while eight had no methylation. In Table 4, the number of cancer samples with aberrant methylation (*n* = 26) and the number of samples without aberrant methylation (*n* = 8) refer to these data.

When methylation ratios were compared between 34 cancer samples in total and 20 normal samples in total, five genes (*HIST1H3J*, *SHOX2*, *PHKG2*, *TLX3*, and *HOXA7*) showed significantly higher methylation in cancer (*P* < 0.05, ranging from 0.0001 to 0.004, *t*-test), and *POU4F2* tended to show higher methylation in cancer (*P* = 0.07, *t*-test) (Figure 7A). Among the 34 cancer samples, 26 showed aberrant methylation in at least one gene, but eight showed no aberrant methylation at all (Figure 7B). When clinicopathological features were compared between methylation(+) cancer and methylation(−) cancer, mutations of *BRAF*/*RAS* oncogenes significantly correlated to methylation(+) groups (*P* = 0.04, Fisher's exact test) (Table 4). Although it was not statistically significant, methylation(+) cancer tended to show larger size of tumor (*P* = 0.06, *t*-test) and higher levels of thyroglobulin (*P* = 0.08, *t*-test).

**Table 4:** **Aberrant methylation and clinicopathological features**.

**Clinical features**	**All Cases (*n* = 34)**	**Aberrant Methylation(+) (*n* = 26, Figure 7B)**	**Aberrant Methylation(−) (*n* = 8, Figure 7B)**	***P*-values**
**SEX**				
Male/female	11/23	10/16	1/7	0.17 (Fisher)
**AGE (YEAR)**				
Mean ± SE	56.0 ± 2.7	57.2 ± 3.1	52.4 ± 4.9	0.45 (*t*-test)
**TUMOR SIZE (mm)**				
Mean ± SE	26.2 ± 2.6	28.3 ± 3.3	20.1 ± 2.0	0.06 (*t*-test)
**NUMBER OF LYMPH NODES WITH METASTASIS**				
Mean ± SE	2.6 ± 0.7	2.2 ± 0.6	3.3 ± 1.6	0.53 (*t*-test)
**DISTANT METASTASIS**				
(+)/(−)	0/34	0/26	0/8	1 (Fisher)
**RECURRENCE**				
(+)/(−)	5/28	3/22	2/6	0.37 (Fisher)
Unknown	1	1	0	
**THYROGLOBULIN (ng/ml)**				
Mean ± SE	104.6 ± 52.1	129.3 ± 68.6	30.5 ± 9.3	0.08 (*t*-test)
**MUTATION OF BRAF/RAS ONCOGENES**				
(+)/(−)	26/7	22/3	4/4	0.04^*^ (Fisher)
Unknown	1	1	0	

SE, standard error. P-values were calculated to compare methylation(+) group and methylation(−) groups and to analyze the correlation of methylation status to clinicopathological features. Fisher, calculated by Fisher's exact test. t-test, calculated by t-test.

* P < 0.05, which is considered as statistically significant. Mutations of BRAF/RAS oncogenes are thus considered to correlate significantly with methylation(+) groups.

## Discussion

In this study, we performed genome-wide DNA methylation analysis in 14 human papillary thyroid cancer samples and 10 normal samples. Although papillary thyroid cancer apparently involves fewer aberrantly methylated genes than other types of cancers, we detected 25 genes frequently hypermethylated in papillary thyroid cancer. Methylation status was quantitatively validated in six out of the 25 genes by pyrosequencing, using the genome-widely analyzed samples and additional samples. Gene silencing in papillary thyroid cancer cell lines was confirmed by real-time RT-PCR. While a subset of cancer cases had no aberrant methylation at all, cancer with preferential methylation tended to have oncogene mutation and to be larger tumor.

Papillary thyroid cancer displayed fewer aberrantly methylated genes, compared with other cancer types (Figure 2). For genes previously reported to be methylated in thyroid cancer, such as *TSHR* (Xing et al., 2003), or in other cancer types, such as *RASSF1A*, *RAR*-β*2*, *p16*, *CDH1*, *DAPK*, and *MLH1*, the methylation frequency in papillary thyroid cancer ranges from 15 to 33% (Hoque et al., 2005; Guan et al., 2008; Mohammadi-asl et al., 2011). In these reports, no or few normal samples were analyzed (Guan et al., 2008; Mohammadi-asl et al., 2011), methylation was also detected in normal samples (Hoque et al., 2005), or a non-quantitative method, i.e., standard methylation-specific PCR, was used (Guan et al., 2008). Standard methylation-specific PCR (Herman et al., 1996) can amplify and detect minor fraction of methylated alleles, but its high sensitivity can lead to overestimation of methylation frequency. Our analysis did not select these genes as frequently methylated ones in the first 14 cancer samples, because normal thyroid tissues also showed high methylation levels or because methylation frequencies in papillary thyroid cancer samples were low (≤2 of 14 cancer samples). Instead, we detected 25 novel genes that were frequently aberrantly methylated (β > 0.25) in at least three of the 14 cancer samples, and not methylated in any of the 10 normal thyroid samples (β < 0.2).

Interestingly, three of the 14 papillary thyroid cancer samples showed no aberrant methylation in the 25 genes, but the other 11 cancer samples showed hypermethylation in at least three of the 25 genes. No cancer sample showed aberrant methylation in just one or two genes. This unusual distribution of aberrant methylation is similar to the CpG island methylator phenotype (CIMP), which was first proposed in colorectal cancer (Toyota et al., 1999). As Toyota et al. demonstrated in colorectal cancer (Toyota et al., 1999), we calculated probability of methylation distribution in papillary thyroid cancer using these 25 genes. The fraction of methylated tumors in each gene was 3/14 for *ATP8A2*, 3/14 for *C19orf4*, …, 5/14 for *ANKRD45*, …, 6/14 for *MYLK*, …, 4/14 for *NRN1*, …, 5/14 for *SHOX2*, and 3/14 for *SST* (Figure 3). Assuming that methylation of these genes is random, the probability that none of the 25 genes would be methylated in three cancer samples is *P* = 1.2 × 10^−8^. This was calculated using the following formula:

$$p(x)=\frac{\left(\begin{array}{l} N\\ x \end{array}\right)\prod_{g}\left(\begin{array}{l} N-x\\ f(g) \end{array}\right)}{\prod_{g}\left(\begin{array}{l} N\\ f(g) \end{array}\right)}$$

where *x* indicates number of samples which do not have methylated genes (*x* = 3 in the present case), *N* indicates number of cancer samples (*N* = 14 here), *g* indicates one of the 25 genes, and *f(g)* indicates the number of samples in which *g* is methylated. Similarly, the probability that at least three of the 25 genes are methylated in 11 cancer samples would be *P* = 0.0028. In each case, a random event is highly unlikely. We rather observe that the associated methylation or un-methylation events both occur at relatively high frequencies. Our data thus suggest that there are two distinct classes of papillary thyroid cancer. One is a subset of hardly methylated cancer. The second one is a subset of preferentially methylated cancer, prone to transcriptional silencing and with the potential to inactivate several genes simultaneously, as CIMP has been proposed in colorectal cancer and other cancers (Toyota et al., 1999; Kaneda et al., 2002; Noushmehr et al., 2010).

Although the number of analyzed samples was not large, preferentially methylated papillary thyroid cancer showed mutation of *BRAF*/*RAS* oncogenes more frequently than methylation(−) cancer (*P* = 0.04, Fisher's exact test). In previous studies of papillary thyroid cancer, although methylation of *RASSF1A* and *BRAF* mutation were detected in a mutually exclusive manner (Xing et al., 2004; Hoque et al., 2005), methylation of *RAR*-β*2* or *MLH1* significantly correlated to *BRAF* mutation (Hoque et al., 2005; Guan et al., 2008). Correlation of aberrant methylation and oncogene mutation are also reported in colorectal cancer; high-methylation and intermediate-methylation epigenotypes strongly correlated to *BRAF* mutation and *KRAS* mutation, respectively, and low-methylation epigenotype strongly correlated to lack of oncogene mutation (Shen et al., 2007; Yagi et al., 2010; Hinoue et al., 2012). The mechanism of these correlations is still unknown, but oncogene mutation may somehow induce aberrant methylation, or aberrant methylation may help escape from senescence by disrupting factors critical in *RAF*/*RAS*-induced senescence (Kaneda and Yagi, 2011).

Preferentially methylated cancer also tended to have larger tumors and higher thyroglobulin levels, which might relate to cancer progression (Piccardo et al., 2013). Although 90% of papillary thyroid cancers are considered to be at low risk with a mortality rate of 1–2%, the mortality rate of the high risk group is 50–75% (Hay et al., 1993; Noguchi et al., 1994; Shaha et al., 1996; Dean and Hay, 2000). The tumor-node-metastasis (TNM) classification is a tool for cancer prognosis; each variable used in TNM staging (age, tumor size, extent of primary tumor, and presence of nodal or distant metastases) shows significant association with observed end points of cancer recurrence or death. Cancer recurrence and mortality ratios are significantly lower in stage I (15.4% and 1.7%, respectively) compared with more advanced tumors (22% and 15.8% in stage II, 46.4% and 30% in stage III, and 66.7% and 60.9% in stage IV, respectively) (Loh et al., 1997). Molecular diagnostic markers are still not used, although their development is anticipated (McLeod et al., 2013). Although aberrant methylation was not significantly associated with lymph node metastasis, distant metastasis, or recurrence in analysis of the 34 cancer samples in this study, further study should be performed using larger set of clinical samples for comparison of aberrant methylation, gene mutation status, and prognosis.

As for detected genes, *TLX3* (*HOX11L2*) is a transcription factor highly expressed in T-cell leukemia (Baak et al., 2008), and its aberrant methylation was observed in cisplatin-resistant bladder cancer (Tada et al., 2011). *SHOX2* is a member of the homeobox gene family, and is reported to relate to a short-stature phenotype of Turner syndrome (Clement-Jones et al., 2000). DNA methylation of *SHOX2* was suggested to be a biomarker for diagnosis of lung cancer based on bronchial aspirates (Schmidt et al., 2010). *HOXA7* is also a transcription factor belonging to the homeobox gene family that regulates gene expression, morphogenesis, and differentiation (La Celle and Polakowska, 2001). *POU4F2* is one of POU family genes with Pit-Oct-Unc domain, and is a transcription factor with a role in cell identity and regulation of nerve cell or retinal development (Weishaupt et al., 2005). PHKG2 is the gamma subunit of phosphorylase kinase, containing the active site of the enzyme. Phosphorylase kinase-deficient liver glycogenosis can be caused by mutations of phosphorylase kinase subunits, *PHKA2*, *PHKB*, or *PHKG2*, but *PHKG2* mutation was reported to cause a severe phenotype of this disease (Burwinkel et al., 2003). *HIST1H3J* encodes a member of histone H3 family, and is found in the small histone gene cluster on chromosome 6p22-p21.3 (NCBI gene data bank). If the role of histone modifications is known to affect the regulation of gene expression, less is known about the possible direct involvement of histones, an H3 variant in the present case, in thyroid tumorigenesis. Further investigation is necessary to clarify tumorigenic roles of these genes and their methylation, in papillary thyroid cancer and other types of cancer (Schmidt et al., 2010; Tada et al., 2011).

In summary, 25 new genes were found to be frequently methylated in papillary thyroid cancer. There might be subsets of papillary thyroid cancer hardly methylated and preferentially methylated, and aberrant methylation of these genes correlates *a priori* to *BRAF*/*RAS* oncogene mutation in papillary thyroid cancer.

## Author contributions

Yasuko Kikuchi, Koichi Yagi, and Keisuke Matsusaka performed the experiments. Eiichi Tsuji and Toshihisa Ogawa prepared clinical samples and information. Junichi Kurebayashi established and supplied cell lines. Yasuko Kikuchi, Shingo Tsuji and Atsushi Kaneda analyzed and interpreted the data. Yasuko Kikuchi and Atsushi Kaneda wrote the manuscript. Toshihisa Ogawa, Hiroyuki Aburatani, and Atsushi Kaneda supervised the study.

### Conflict of interest statement

The authors declare that the research was conducted in the absence of any commercial or financial relationships that could be construed as a potential conflict of interest.
